# Commensal bacteria-derived extracellular vesicles suppress ulcerative colitis through regulating the macrophages polarization and remodeling the gut microbiota

**DOI:** 10.1186/s12934-022-01812-6

**Published:** 2022-05-16

**Authors:** Liping Liang, Chenghai Yang, Le Liu, Genghui Mai, Haolin Li, Lele Wu, Ming Jin, Ye Chen

**Affiliations:** 1 Department of Gastroenterology, State Key Laboratory of Organ Failure Research, Guangdong Provincial Key Laboratory of Gastroenterology, Nanfang Hospital, Southern Medical University, 1838 North Guangzhou Road, Guangzhou, 510515 China; 2 Integrative Microecology Center, Department of Gastroenterology, Shenzhen Hospital, Southern Medical University, Shenzhen, 518100 China

**Keywords:** Ulcerative colitis, Extracellular vesicles, *Clostridium butyricum*, Gut microbiota, Macrophage polarization

## Abstract

**Background:**

The extracellular vesicles (EVs) traffic constitutes an essential pathway of cellular communication. And the molecules in EVs produced by procaryotes help in maintaining homeostasis, addressing microbial imbalance and infections, and regulating the immune system. Despite the fact that *Clostridium butyricum* (*C. butyricum*) is commonly used for treating ulcerative colitis (UC), the potential role of *C. butyricum*-secreted EVs in commensals-host crosstalk remains unclear.

**Results:**

Here, we performed flow cytometry, western blot, immunohistochemistry and 16S rRNA analysis to explore the role of *C. butyricum*-derived EVs on macrophage polarization and gut microbiota composition in a dextran sulfate sodium (DSS)-induced UC mouse model. The antibiotic cocktail-induced microbiome depletion and faecal transplantations were used to further investigate the mechanisms by which EVs regulate macrophage balance. Our findings showed that *C. butyricum*-derived EVs improved the remission of murine colitis and polarized the transformation of macrophages to the M2 type. Furthermore, *C. butyricum*-derived EVs restored gut dysbiosis and altered the relative abundance of *Helicobacter, Escherichia-Shigella, Lactobacillus*, *Akkermansia* and *Bacteroides,* which, in turn, faecal transplantations from EVs-treated mice relieved the symptoms of UC and improved the impact of EVs on the reprogramming of the M2 macrophages.

**Conclusion:**

*C. butyricum*-derived EVs could protect against DSS-induced colitis by regulating the repolarization of M2 macrophages and remodelling the composition of gut microbiota, suggesting the potential efficacy of EVs from commensal and probiotic *Clostridium species* against UC.

**Supplementary Information:**

The online version contains supplementary material available at 10.1186/s12934-022-01812-6.

## Introduction

In general, inflammatory bowel disease (IBD) contains genetic disorders caused by chronic inflammation in the digestive tract, such as ulcerative colitis (UC) and Crohn's disease (CD). Although IBD has an elevated rate of incidence and has been extensively studied, its pathophysiology has remained unknown. Previous researches have indicated that the onset of IBD might be potentially associated with several hereditary factors, immunity, gut microbiota, cellular apoptosis, and susceptibility to infections [1]. Most of the therapeutic drugs used for treating IBD, such as corticosteroids, immunosuppressive agents, 5-aminosalicylic acid, and antibiotics, as well as surgical treatment, in extreme cases, present severe side effects including allergic response and intolerance. Processing with anti-tumor necrosis factor-α (TNF-α) antibody has resulted in an improved remission maintenance rate; however, the dosage effect, increased susceptibility to infections, and development of neutralizing antibodies still present a challenge. One of the preferred therapies for inducing and maintaining remission of UC involves enteral nutrition (EN); however, EN alone has been effective in only 30–78% of patients [2]. Thus, there is an urgent need to develop novel treatment strategies for UC.

Previous researches have explained that UC progression is correlated with an increase in the M1 macrophage inflammation-inducing population, along with a decrease in the M2 macrophage, M1-inhibitory population [3]. Additionally, in patient with UC, the dysfunctional gut microbiome increases the intestinal epithelial cell barrier permeability, enhancing intestinal inflammation. Furthermore, UC patients exhibit a decreased level of commensal bacteria with probiotic properties, which play an immunoregulatory role in promoting intestinal mucosal repair [4]. Thus, regulating the gut microbiome to repair intestinal mucosa holds clinical significance in UC. The composition of the colonic microbiota, which is vital for human health, is dependent on its potential to access dietary nutrients. Despite reports on the direct link between gut microbial dysbiosis and IBD, a direct causal relationship cannot be assumed between them. Also, investigations have illustrated that the gut microbiome composition is affected by intestinal inflammation. The innate immune system is composed of macrophages that have pattern recognition receptors to identify foreign bacteria and perform phagocytosis [5]. In a non-diseased state, the commensal bacteria induce the immune response, whereas in diseased state, such as UC and CD, the inflammatory macrophages are involved in eliciting the immune response [6]. Patients with IBD harbor adherent-invasive *E.coli* (AIEC), which invade the intestinal epithelial cells, and are ultimately engulfed by macrophages, where they undergo replication due to autophagy defects, resulting in IBD [7]. The anti-inflammatory effect of short-chain fatty acids (SCFAs), such as the butyrate, regulates the intestinal macrophage function via an inhibitor of histone deacetylase or suppression of the activation of NF-κB [8]. Furthermore, butyrate promotes IL-10 expression to exhibit anti-inflammatory activity [9]. Although macrophages are involved in the crosstalk between IBD and gut microbiota, not much is known regarding the relevant mechanism of action.

The gut microbiota uses various mechanisms to interact with the host cells. One of them involves the generation of membrane vesicles that are used to transfer the cargo over a large distance in the host [10]. Microbiota-derived extracellular vesicles (EVs) are known to facilitate the transfer of active compounds between the cells [11]. Bacteria-derived EVs interact with host cells via pattern recognition receptors to activate the signalling pathways to alter the activity of cytokines/chemokines [12, 13]. Additionally, bacteria-derived EVs also promote the horizontal transfer of antibiotic-resistant genes to the surrounding bacteria [14]. They also facilitate detoxification to promote bacteria survival. A study showed that commensal bacteria-generated EVs promoted their colonization in the gastrointestinal tract, thus, supporting that bacterial EVs favoured bacterial adaptation to a novel niche [15]. Several studies have analyzed the healing ability of specific probiotic strains to treat IBD. The results have indicated that bacteria affect gut performance by increasing gut permeability/histological modifications and decreasing the secretion of inflammatory cytokines. *Clostridium* *butyricum* (*C. butyricum*) has been used effectively for maintaining the remission in a murine model of dextran sodium sulfate (DSS)-induced colitis [16]. On the other side, there is only one clinical research that manifested the beneficial effect of *C. butyricum* in maintaining remission in UC patients with food allergies [17]. Studies should also be done to analyze the strategies involved in the advancement and release of secreted bacterial factors in such diseases. *C. butyricum*-derived EVs are known to perform a critical task in bacteria-host interactions compared with other discharged elements, promoting the transfer of effector molecules across the host cells [18]. Thus, we hypothesized that in UC, *C. butyricum*-derived EVs could have an immunomodulatory effect on the M1/M2 equilibrium by regulating the gut microbiota. Here, we first investigated the possibility of *C. butyricum*-derived EVs-induced M2 macrophage polarization in a mice model of UC. Next, we performed 16S rRNA analysis post-*C. butyricum*-derived EVs treatment to evaluate the changes in the composition of the gut microbiome. Further, *C. butyricum*-derived EVs-mediated regulation of the M2 macrophage differentiation was confirmed by performing faecal transplantations to prevent UC by regulating the gut microbiome.

## Materials and methods

### Bacterial culture

*C. butyricum* strain MIYAIRI 588 (CBM588) was obtained from Miyarisan Pharmaceutical Co. Ltd, Tokyo, Japan and was cultured in reinforced clostridial medium (Solaribio, China) or in tryptone–peptone–glucose–yeast extract (TPGY) broth (pH = 7.0) under anaerobic conditions (10% H_2_, 80% N_2_, and 10% CO_2_) at 37 °C.

### Isolation, purification, characterization of EVs

After pelleting of bacterial cultures (10,000×*g* for 20 min), the obtained supernatants were filtred (0.22 μm) to remove parental bacterial debris and other impurities. To get the crude EVs, the supernatant was further concentrated to 1/8 of its initial volume using 100 kDa ultrafiltration membranes (Millipore, Germany) and ultracentrifuged at 4 °C and 60,000×*g* (Hitachi C21G, HITACHI, Maru, Chiyoda, Tokyo, Japan) for 30 min, and washed with PBS twice. The purified EVs were obtained with filtering (0.22 μm) again before ultracentrifugation in a 45 Ti rotor at 150,000×*g* at 4 °C for 2 h using a sucrose density gradient, followed by using Detoxi-gel endotoxin removing columns (Thermo Scientific, USA) to remove endotoxin [19]. The final pellets were resuspended in PBS and stored at − 80 °C.

To confirm the morphological characteristics of the isolated EVs, transmission electron microscope (TEM) was conducted. Briefly, 15 μl of EV solution was applied on a copper grid for 1 min, and the excess solution was blotted with filter paper. For negative staining of EVs, 2% uranyl acetate (15 μl) was applied to the grid for 1 min, and then blotted with filter paper. Grids were thoroughly dry before imaging under an FEI Tecnai transmission electron microscope (FEI, USA).

To detect the diameter and particle number of the EVs, nanoparticle tracking analysis (NTA) was performed using a ZetaView PMX 110 instrument (Particle Metrix, Germany). For each measurement, the sample was diluted in 1xPBS, and 11 different cell positions were recorded for three cycles under the following settings: shutter: 70; scattering intensity: 4.0; temperature: 25 °C. Polystyrene particles (110 nm) were used to calibrate the instrument before the measurement. Collected data for each sample was analyzed with the corresponding software, ZetaView 8.04.02.

### Animals

Wild-type male C57BL/6 mice (20–24 g; 40–60 days old) purchased from the Laboratory Animal Centre of Southern Medical University (Guangzhou, China) were kept at 21 ± 2 °C, with a relative humidity of 45 ± 10% on a 12 h dark/light cycle with free access to water/food under specific pathogen-free (SPF) barrier conditions. Euthanasia was performed by CO_2_ gas in a closed chamber, followed by cervical dislocation. All animal experiments were performed at the SPF Animal Room, Southern Medical University. The protocols of experiments were confirmed and approved by the Institutional Animal Care and Use Committee of Southern Medical University, China.

### Treatment of animals

Three treatment groups (Control, DSS, and DSS+ EVs) were randomly assigned to six mice each. UC was induced by administering 2% (w/v) DSS (36–50 kDa, Millipore Corporation, Billerica, MA, USA) in the drinkable water for 5 days, succeeded by ordinary water for 5 days. Mice in the DSS+ EVs group received intragastrically 0.2 ml of PBS containing 15 μg of EVs and in the control and DSS groups received 0.2 ml PBS once every day. The treatment dosage of 15 µg EVs was chosen based on previously available studies and a dose–effect relationship experiment on DSS induced colitis model [20–22].

#### For gut microbiome depletion

Mice were administered a blend of the following antibiotics: metronidazole (1.0 g/l); ciprofloxacin (0.2 g/l), dissolved in drinking water for 14 days. Gut microbiota depletion was confirmed by collecting faecal samples and assessing the total DNA. Three groups (Control, antibiotics+ DSS, and antibiotics+ DSS+ EVs) were randomly assigned to six mice each. The control group did not receive any treatment; the groups with antibiotics+ DSS received an antibiotics blend, followed by DSS for UC induction. The antibiotics+ DSS+ EVs group received 0.2 ml of PBS containing 15 μg of EVs intragastrically once every day, whereas mice in the groups with antibiotics+ DSS received 0.2 ml of normal PBS orally.

#### For faecal transplantation assays

Four groups (DSS+ EVs+ , DSS+ EVs−, DSS−EVs+ , and DSS−EVs−) were randomly assigned six mice each. The groups with DSS+ were given DSS for UC induction; the DSS−EVs− group received regular feed; the groups with EVs+ were given oral EVs once every day, and the groups with EVs− were given 0.2 ml PBS treatment. Ten days later, stool samples were collected every day for the next 10 days under sterile conditions. The stool samples from each group were combined to obtain a 100 mg sample, which was resuspended in 1.0 ml of sterile saline, stirred potently for 10 s, followed by centrifugation for 3 min at 800×*g*. The supernatant was used for transplantation. Changes in bacterial composition were prevented by preparing each transplantation sample 10 min before intragastrical gavage. Each recipient group was randomly assigned six mice who were given DSS for UC induction, followed by intragastrical administration of 100 µl of fresh transplant material every day for 10 days.

### Disease activity index (DAI)

The monitoring of disease activity index (DAI) was carried out daily. It comprised of stool consistency, body weight, and stool bleeding. Scoring was done as follows: stool consistency (3–4 = diarrhea; 1–2 = loose stool; and 0 = normal); Weight loss (0 = none; 1 = 1–5%; 2 = 5–10%; 3 = 10–15%; and 4 =  > 15%); Stool bleeding (0 = negative; 1 =  + ; 2 =  ++ ; 3 =  +++ ; and 4 =  ++++) [23].

### Histology

After removal, the spleen, liver, small intestine, kidney, and colon of the mice were fixed using paraformaldehyde, inserted in paraffin, and cut into segments (5 µM). The extent of inflammation in the H&E stained sections was determined based on a histological colitis score: 0 to 14 (maximum score).

#### Ex vivo *and* in vivo imaging

EVs were incubated with 10 µM DiI (Beyotime Biotechnology, Shanghai, China), a lipophilic fluorescent dye, for 30 min at room temperature. DiI-labeled EVs were isolated using ultracentrifugation and then injected orally into C57BL/6 mice. After 8 h, the mice were killed, and gastrointestinal tissues were acquired. DiI fluorescence and EV distribution were detected by the IVIS spectrum.

Intestinal permeability measurement was performed upon oral gavage of FITC-dextran solution (4 kDa, 600 mg/kg; Sigma-Aldrich). After 4 h absorption and excretion, mice were anaesthetized and exposed to the IVIS Spectrum CT system, and the fluorescent retentions of FITC-dextran were measured.

### Isolation of lamina propria mononuclear cells (LPMCs)

The large intestines were opened longitudinally, rinsed with PBS (Gibco, USA) to eliminate fecal matter, cut into 1 cm long strips, followed by stirring in Hanks' Balanced Salt Solution (HBSS, Gibco, USA) containing 1 mM DTT (Fudebio, Hangzhou, China) and 2 mM EDTA at 37 °C for 30 min. The isolation of the cells was performed from the intestinal lamina propria (LP) following a previously described method. In brief, the intestinal tissue was digested using 1 mg/ml of collagenase (type IV), and 5% FBS (Gibco, Grand Island, NY, USA) in RPMI 1640 medium (Gibco, USA) at 37 °C for 30 min with stirring. Every 30 min, we centrifuged the released cells and stored them in the culture medium. Fresh collagenase was added to replace the mucosal pieces at least 2 times. Finally, the purification of the LP cells was carried out utilizing a discontinuous Percoll (Yeasen, Shanghai, China) gradient collecting at the 40–70% interface.

### Flow cytometry

The cellular suspensions of LP were kept at 4 °C for 30 min with anti-CD16/32, anti-F4/80, and anti-CD206 fluorochrome-conjugated antibodies (BD Pharmingen, USA) to evaluate the M1/M2 macrophage equilibrium. Data were collected on a FACSCalibur flow cytometry instrument and analyzed using FlowJo software 7.6 (FlowJo, Ashland, OR, United States).

### Isolation of fecal DNA and 16S rRNA analysis

Total DNA from fresh stool samples was extracted using the E.Z.N.A.^®^ Stool DNA Kit (Omega Bio-tek, GA, USA) and quantified following a previously described technique [24]. Briefly, the V3–V4 regions of the 16S rRNA genes were amplified utilizing the extracted and diluted DNA (1 ng/µl) from each sample as a template. The following primers were used: (806R: 5′-GGACTACHVGGGTWTCTAAT-3′; 338F: 5′-ACT CCTACGGGAGGCAGCAG-3′) in a TransStart^®^ Fastpfu DNA polymerase (TransGen, Beijing, China). A 2% agarose gel electrophoresis was used to detect the products of PCR and purified utilizing the Kit of AxyPrep DNA gel extraction (Axygen Biosciences, CA, USA). Next, sequencing libraries were created utilizing the TruSeq DNA PCR-Free Library Preparation Kit (Illumina, USA) on the Illumina Miseq platform according to the manufacturer's recommendations. The raw sequencing data were quality-filtered using fastp version 0.20.0 [25] and merged using FLASH version 1.2.7 [26]. Raw data were further analyzed by Majorbio Bio-Pharm Technology Co. Ltd. (Shanghai, China).

### Western blotting

The expression of the protein related to MUC2, ZO-1, IL-10, TNF-α, arginase 1(Arg1), inducible nitric oxide synthase (NOS2) (1:1000; Affinity, USA) was evaluated using western blot following a previously described method [27]. Quantification of band intensities expressed the modifications in the relative levels of the specific proteins. ImageJ computer program (National Institutes of Health, Bethesda, MD, USA) was utilized for densitometric studies.

### Immunohistochemical staining

After deparaffinization and rehydration of the paraffin-embedded sections, antigen retrieval was performed following the standard protocol. Next, treatment with 3% H_2_O_2_ was followed by blocking with goat serum for 1 h at 37 °C, succeeded by incubation at 4 °C during the night, with the specific primary antibody. After washing the sections with cold PBS three times, they were incubated with a second antibody (ZSGB-BIO), developed with the DAB reagent and counterstained with haematoxylin. Negative controls were incubated without antibodies.

### Immunofluorescence staining

After deparaffinization using xylene and dehydration using gradient ethanol, the slides were permeabilized. Next, the slides were kept in nightly incubation with the specific primary antibody in 5% normal goat serum at 4 °C. After washing the sections with cold PBS, they were incubated with the secondary antibody of Alexa Fluor 488/594-labelled (Yeasen, Shanghai, China) for 1 h at 25 °C. DAPI (Boster, Wuhan, China). was used to stain the cell nuclei. The sections were observed under the DM4B fluorescence microscope system (Leica, Wetzlar, Germany), and the mean fluorescence intensity of images was analyzed at identical microscopic settings.

### Statistical analysis

A one-way analysis of variance (ANOVA) was utilized for data analysis, succeeded by comparison multiple tests of Newman–Keuls. Data was studied utilizing SPSS version 21.0 (Chicago, IL, USA) and expressed as mean ± standard deviation (mean ± SD). GraphPad Prism 7 (San Diego, CA, USA) was used for curve-fitting. P-values were considered statistically significant < 0.05.

## Results

### *C. butyricum*-derived EVs ameliorated DSS-induced UC in mice

**Fig.1:**
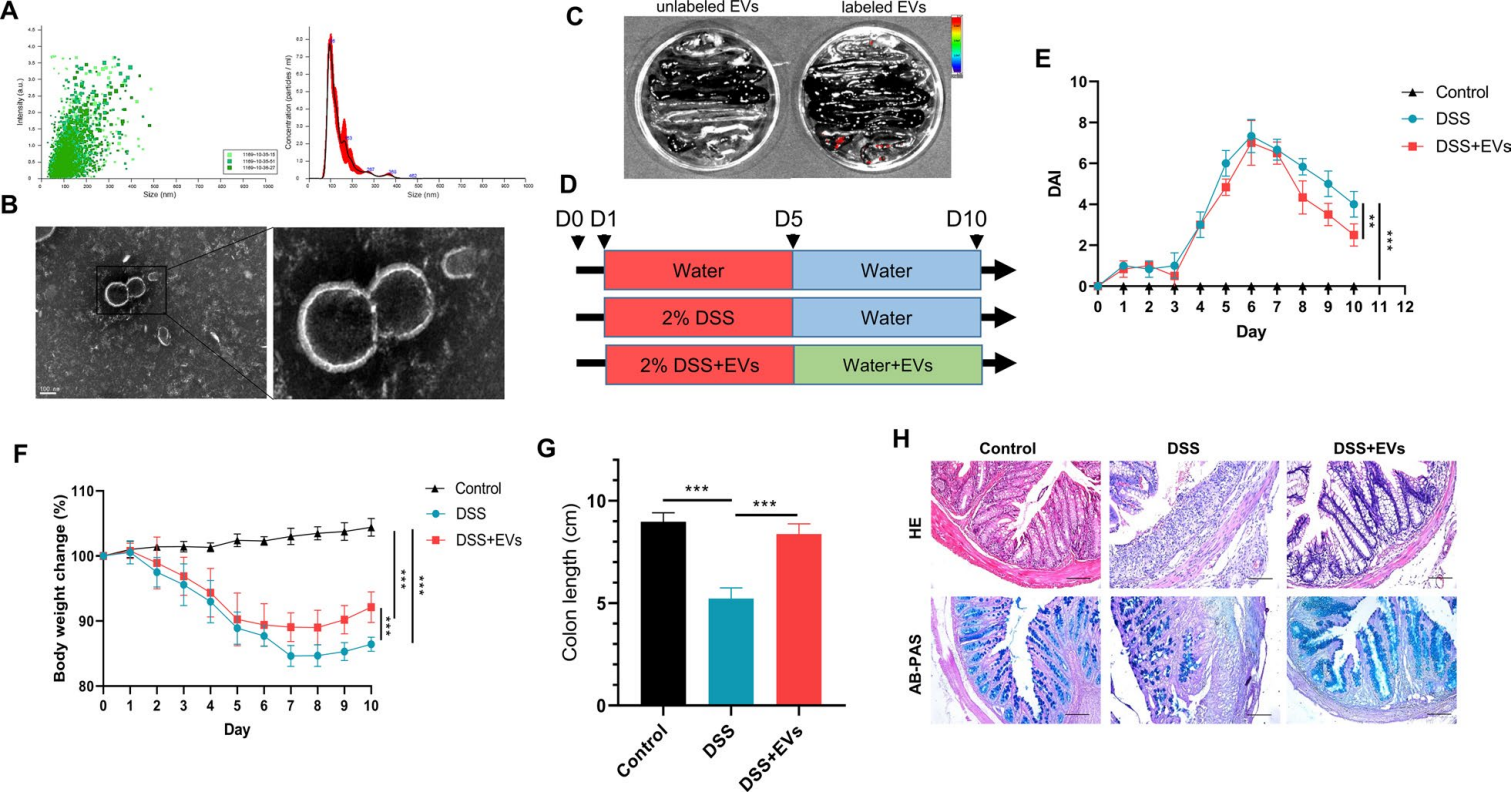
*C. butyricum*-derived EVs suppressed the progression of acute DSS-induced colitis. **a** Nanoparticle tracking analysis (NTA) for characterization of *C. butyricum*-derived EVs. **b** Transmission electron microscopy images of isolated EVs. Scar bar was 100 nm. **c** Ex vivo image of gastrointestinal tract, after 8 h of EVs feeding. **d** Mice were given 5 days of 2% DSS treatment to induce acute colitis, followed by intragastrical administration of *C. butyricum*-derived EVs in the next 5 days, or treated with drinking water as control (n = 6 per group). **e** DAI, **f** body weight change, and **g** colon length was measured at the indicated time points. **h** H&E and AB-PAS staining of each treatment group. Scar bars were 100 μm, respectively. Data are presented as means ± SD, n = 6 mice per group. *P < 0.05; **P < 0.01; ***P < 0.001

**Fig.2:**
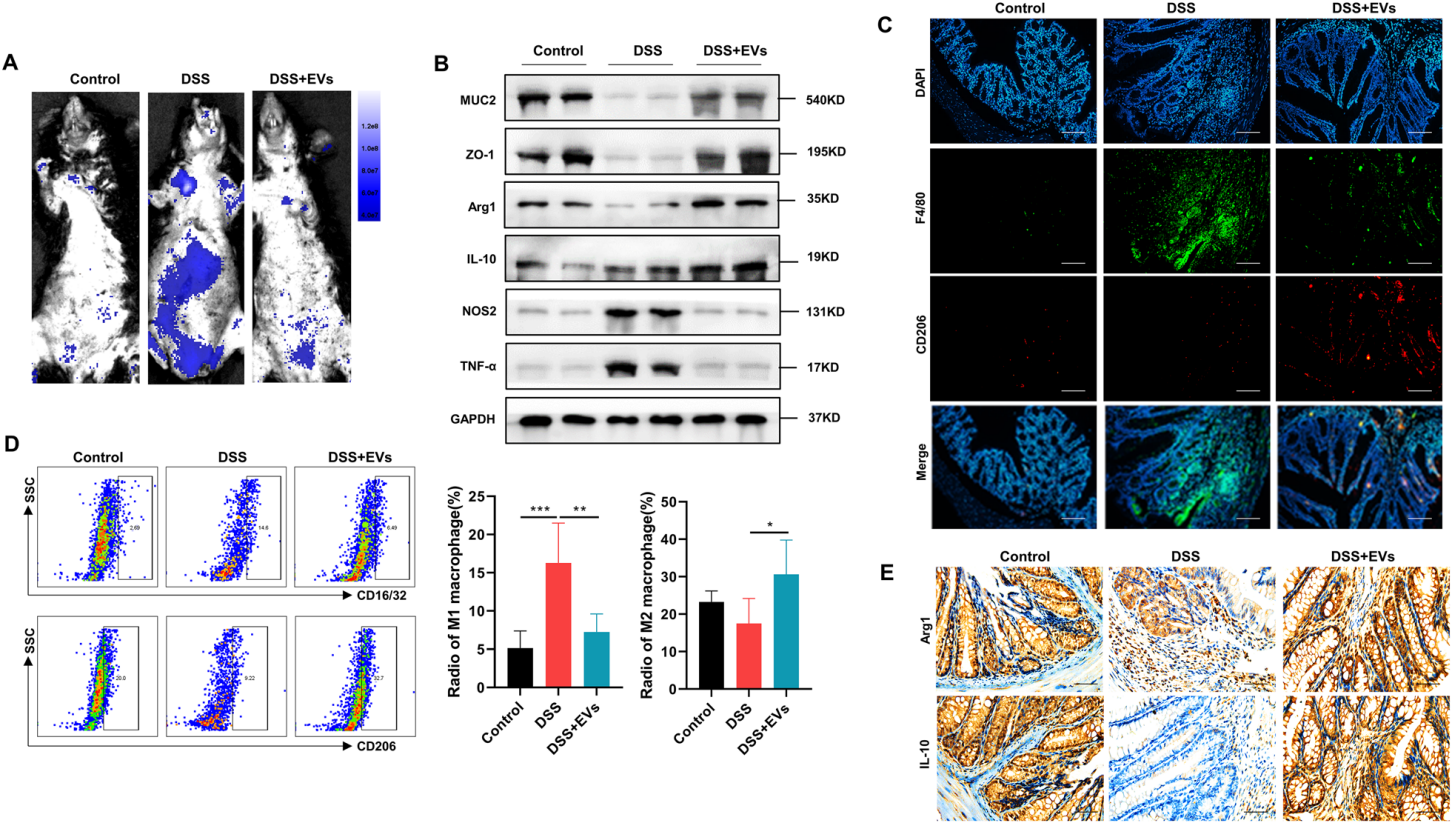
*C. butyricum*-derived EVs improved M2 macrophage polarization after DSS challenge. **a** Intestinal permeability measurement by intragastrical administration of FITC-dextran (600 mg/kg). **b** Expression of barrier-associated proteins (MUC2 and ZO-1), M2 macrophage markers (Arg1 and IL-10), and M1 macrophage markers (NOS2 and TNF-α) were measured by Western blotting. **c** Representative immunofluorescence staining of F4/80 (macrophage markers) and CD206 (M2 macrophage markers). Scar bars were 100 μm. **d** LPMCs were isolated and determined by flow cytometry; CD16/32 and CD206 were used as the marker of M1 and M2 macrophages, respectively. The ratio of M1 and M2 macrophages was shown on the right panel. **e** Colon tissues were stained by immunohistochemistry for Arg1 and IL-10 as M2 macrophage markers. Scar bars were 50 μm. Data are presented as means ± SD, n = 6 mice per group. *P < 0.05; **P < 0.01; ***P < 0.001

*C. butyricum*-derived EVs were obtained from the culture supernatants of *C. butyricum* using a series of filtration and centrifugation steps. These EV-containing fractions were mainly characterized by NTA analysis, which showed that the sizes of EVs ranged from 30 to 200 nm (Fig. 1a), and TEM images also exhibited a nano-sized vesicle with a lipid bilayer (Fig. 1b). Together, these results demonstrate that *C. butyricum* can release nano-sized vesicles. To understand the mechanism of action of EVs in regulating intestinal barrier functions, we checked the distribution of DiI*–*labeled *C. butyricum*-derived EVs in gastrointestinal tissues. The result suggest that most of the EVs can translocate into the large intestine within 8 h of feeding (Fig. 1c). Based on the previously known immunomodulatory functions of *C. butyricum *[28], we explored *C. butyricum*-derived EVs therapeutic role in a mice model of UC (Fig. 1d). Post-treatment with DSS, mice showed a high DAI score along with acute weight loss, rectal bleeding, diarrhoea, and reduced colon length. Expectedly, *C. butyricum*-derived EVs prevented the increase in DAI (Fig. 1e) and weight loss (Fig. 1f), improved colon length (Fig. 1g), thus, inhibiting disease progression and a reduced mortality rate compared with the control group. Histologic analysis revealed DSS-treatment induced crypt distortion, epithelial injury, loss of goblet cells, and inflammatory cell infiltration in the mucosa and submucosa of mice with UC, which were considerably reversed by *C. butyricum*-derived EVs treatment (Fig. 1h). Next, mice were administered FITC-dextran through oral gavage 4 h before being euthanized to evaluate the impact of *C. butyricum*-derived EVs on intestinal permeability in UC. The results showed that a significant enhancement in the serum fluorescence intensity of FITC-dextran post-treatment with DSS, which were attenuated by *C. butyricum*-derived EVs, compared with the control group (Fig. 2a). Consistently, by contrast to control mice, significant decreases in colonic mucus and tight junction proteins (MUC2 and ZO-1) were observed in DSS-colitis mice, and *C. butyricum*-derived EVs upregulated the expression level of gut barrier related-proteins **(**Fig. 2b). Thus, *C. butyricum*-derived EVs protected the mice against DSS-induced colitis by lowering inflammation, reducing tissue damage, and maintaining intestinal functionality and integrity.

### *C. butyricum*-derived EVs improved M2 macrophage polarization in DSS-induced UC in mice

Macrophages are known to exist in a reversible equilibrium state between the pro-inflammatory M1 type and the anti-inflammatory (healing) M2 type and can transform from M1 to M2 type in cases of tissue injury/infection, including UC. Thus, we evaluated if *C. butyricum*-derived EVs could alter this equilibrium in favor of the M2 type, which would induce elevated levels of anti-inflammatory cytokines. Western blot analysis of the DSS-treated colon tissues revealed downregulated protein expression of M2 genes, including Arg1 and IL-10, indicating depleted levels of M2 macrophages in mice with DSS-induced UC. On the contrary, *C. butyricum*-derived EVs treatment resulted in considerably elevated colonic expression of M2 genes compared with the control groups. Next, in the DSS group, we observed increased NOS2 expression and elevated TNF-α expression compared with the control group, which were reversed in the DSS+ EVs group (Fig. 2b). Thus, treatment with *C. butyricum*-derived EVs altered the equilibrium in favor of the anti-inflammatory M2 type macrophages in DSS-exposed colons, which provided relief from the symptoms of UC. The results of immunofluorescence for F4/80 and CD206 show the same trend (Fig. 2c). Additionally, we used flow cytometry with F4/80 and CD16/32 or CD206 (macrophage markers) stainings to test macrophages infiltration and differentiation. We found that the elevated count of the activated F4/80+ and CD16/32+ macrophages in DSS group was remarkably decreased post-processing with *C. butyricum*-derived EVs. The DSS group showed an elevated count of the cells of M1 (F4/80+ CD16/32+) and a depleted count of M2 (F4/80+ CD206+) cells compared with the control group (Fig. 2d). However, *C. butyricum*-derived EVs treatment skewed this ratio by increasing the proportion of the cells of M2 (F4/80+ CD206+) and reducing the proportion of the cells of M1 (F4/80+ CD16/32+) in the gut (Fig. 2d). Thus, *C. butyricum*-derived EVs suppressed macrophage infiltration and induced the polarization of M2 macrophages in the gut of mice with DSS-induced UC. The results of immunohistochemistry for Arg1 and IL-10 show the same trend (Fig. 2e). Thus, all data suggested that *C. butyricum*-derived EVs significantly protected DSS-induced intestinal damage via regulating the polarization of M2 macrophages.

### *C. butyricum*-derived EVs modified the gut microbiota composition in UC mice

**Fig.3:**
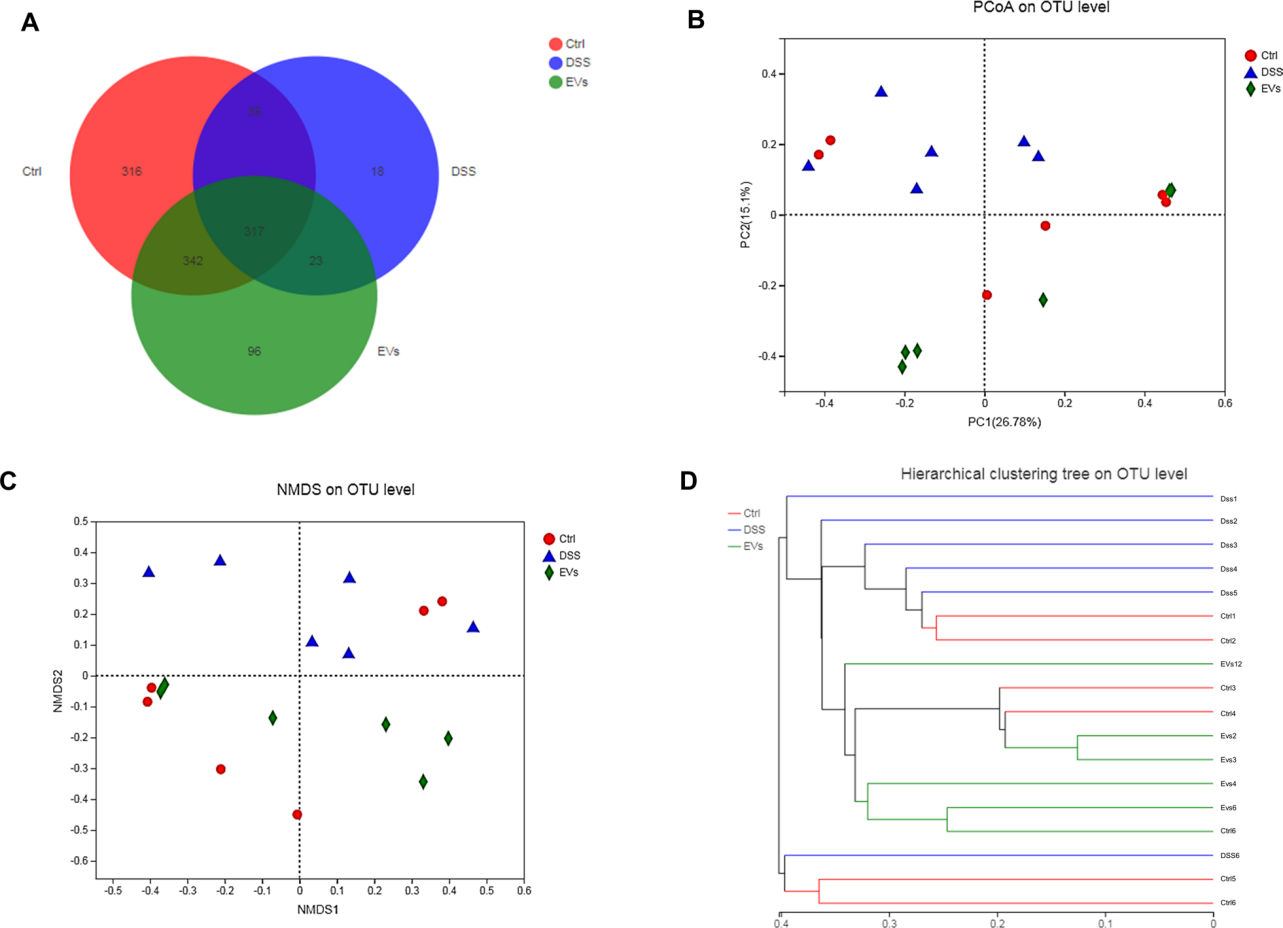
Effects of *C. butyricum*-derived EVs on overall structural modulation of gut microbiota in DSS-induced colitis mice. **a** Venn diagram showed the differential numbers of OTUs in each group. **b** PCoA analysis based on unweighted UniFrac distances indicated a prominent site-specific clustering with the different groups. **c** NMDS analysis. **d** Hierarchical clustering tree of gut microbiota based on weighted Unifrac metrics indicated the different beta diversity of gut microbiota in each group

The modifications in the gut microbiota composition were studied after treatment with *C. butyricum*-derived EVs using 16S rRNA sequencing. Eighteen samples generated 1, 243 OTUs and 879, 082 useable reads. The Control and DSS groups contained 1055 OTUs; the DSS+ EVs and control groups contained 1133 OTUs; the DSS+ EVs and DSS groups contained 835 OTUs; and 317 overlapping OTUs were discovered by constructing a Venn diagram between these three groups (Fig. 3a). There was no significant difference in Shannon index among the three groups (Additional file 1: Figure S1). We observed different gut microbiome in the DSS and the control groups by PCoA (principal co-ordinates investigations) with lesser distances among the control and DSS+ EVs groups compared to the Control and DSS groups (Fig. 3b). NMDS analysis of the intestinal flora also showed a clear difference (Fig. 3c). Hierarchical clustering analysis of the OTUs revealed that a significant distance was found in each group with a small distance among the DSS+ EVs and the Control groups, which indicated that the microbial community structure was significantly different between the Control groups and DSS groups, and the composition of bacteria within groups was similar (Fig. 3d).

**Fig.4:**
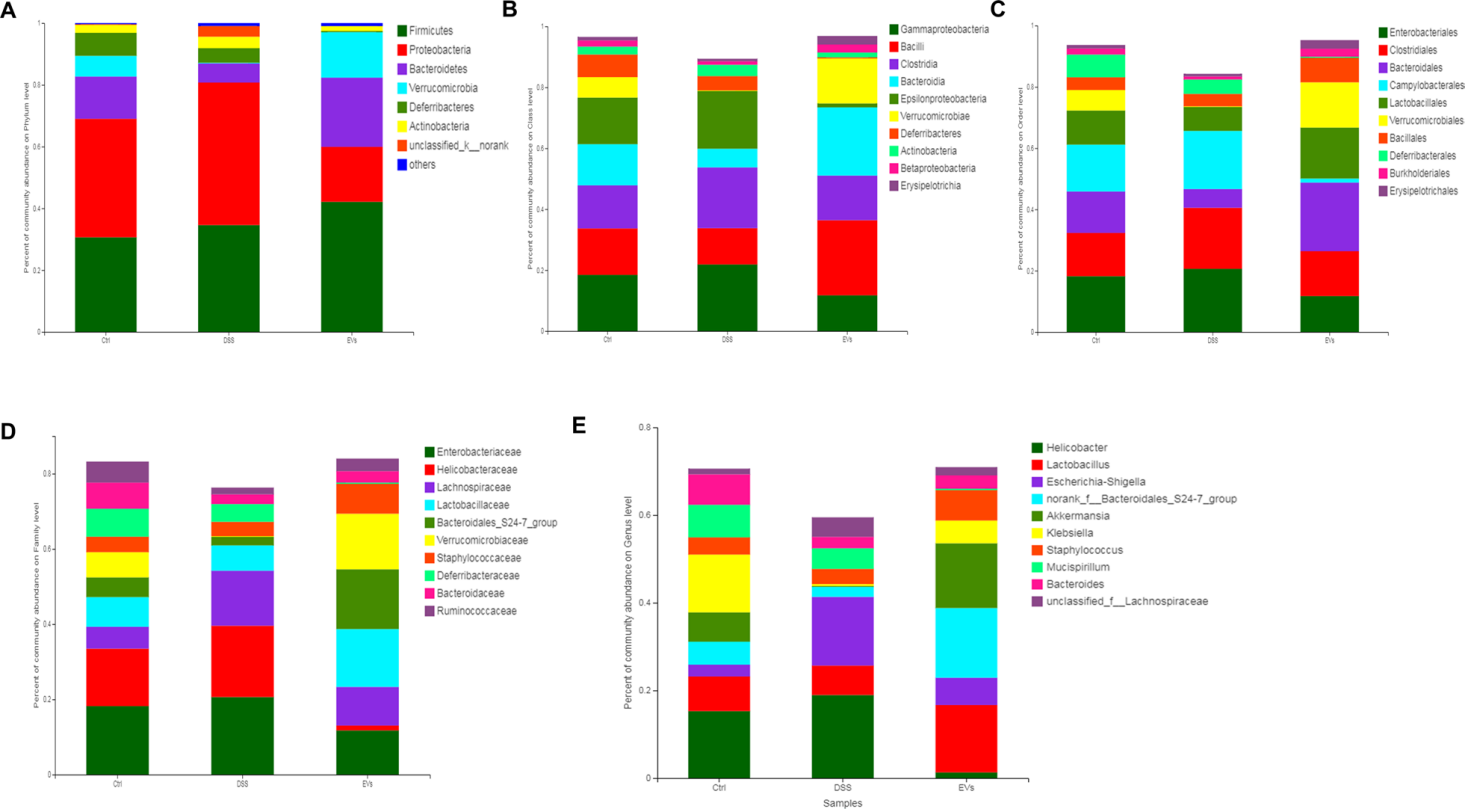
*C. butyricum*-derived EVs modulated the microbial composition in DSS-induced colitis. **a** Phylum. **b** Class. **c** Order. **d** Family. **e** Genus

Next, the gut microbiota community structure was reported using histograms at the phylum, class, order, family, and genus levels. All samples contained abundant *Bacteroidetes, Firmicutes, and Proteobacteria*. Compared to the control group, DSS decreased the relative abundance of *Bacteroidetes* and increased the levels of *Firmicutes* and *Proteobacteria *(Fig. 4a). Treatment with *C. butyricum*-derived EVs reduced these DSS-induced changes. Fourteen classes including *Gammaproteobacteria, Bacilli, Clostridia*, *Bacteroidia,* and *Epsilonproteobacteria* were found in all samples (Fig. 4b). The results showed that the relative abundance of *Bacilli*, *Bacteroidia and Verrucomicrobiae* drastically dropped in the DSS group, but increased significantly following treatment with EVs. Sequencing data identified 18 orders of gut microbes (Fig. 4c). *Bacteroidales*, *Lactobacillales*, and *Verrucomicrobiales* were expressed at high levels in the EVs group, but at low levels in the DSS group. Sequencing data identified 23 families of microbial flora, which is similar to the observations made at the phylum and class levels. *Enterobacteriaceae, Helicobacteraceae,* and *Lachnospiraceae* were dominant communities in the DSS group, but were reduced by EVs treatment. Compared with the control group, *Bacteroidales*_S24-7_group and *Verrucomicrobiaceae* were observed at much lower levels in the DSS group, and EVs protected against DSS-induced downregulation of these families **(**Fig. 4d). Finally, 33 genera were identified in all samples. The relative abundances of *Lactobacillus*, *Bacteroidales*_S24-7_group*, Akkermansia* and *Bacteroides,* were significantly down-regulated in response to DSS treatment, and EVs treatment reversed these decreases (Fig. 4e).

### Modulation of M1/M2 equilibrium by *C. butyricum*-derived EVs following gut microbiome depletion

**Fig.5:**
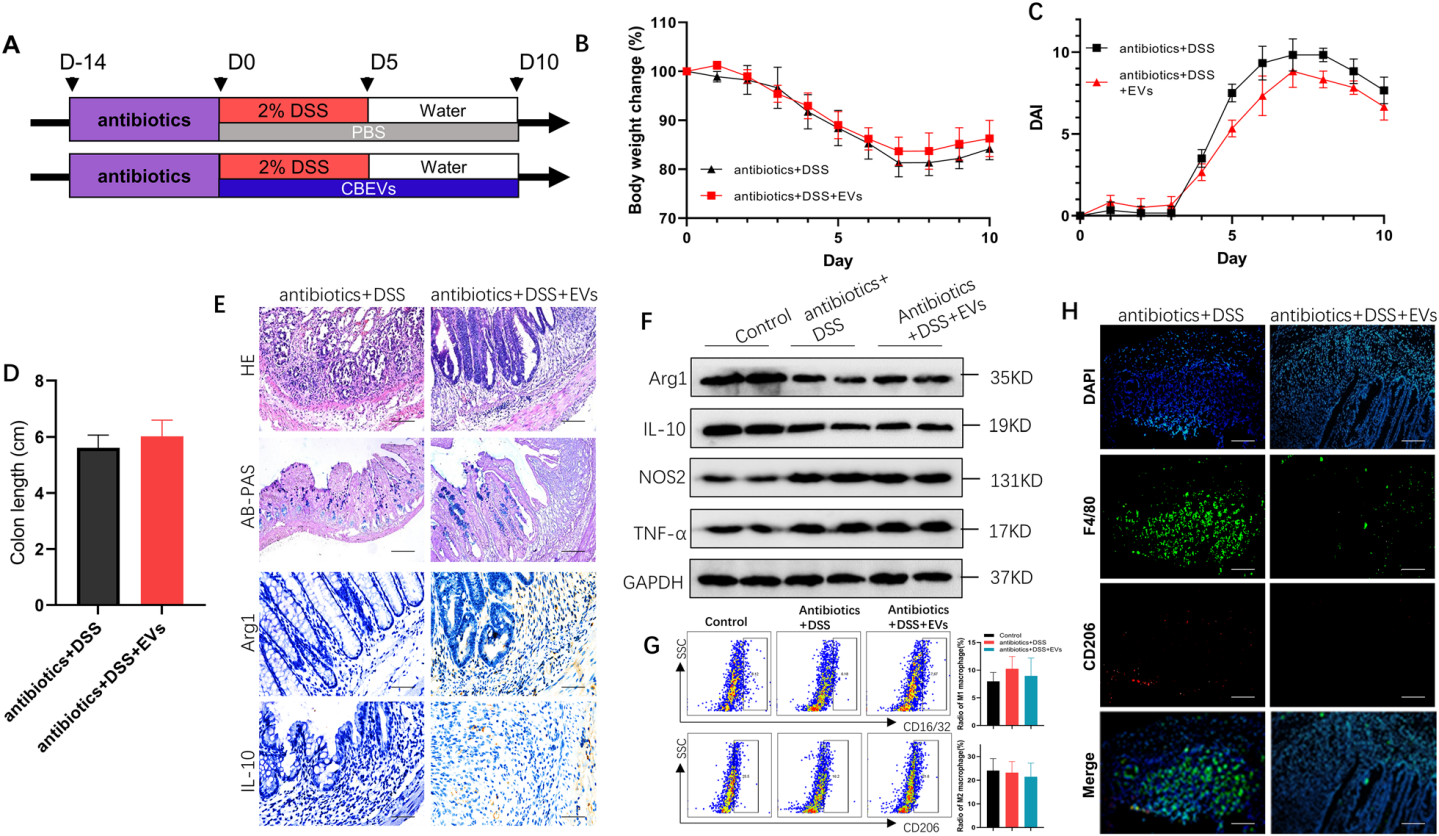
Antibiotics application impaired the promotive effect of *C. butyricum*-derived EVs on M1/M2 macrophage balance. **a** Mice were treated with antibiotics to deplete the gut microbiota for 14 consecutive days, followed by oral administration of DSS in the presence or absence of *C. butyricum*-derived EVs in the next 5 days, and were euthanized 5 days later (n = 6 per group). **b** body weight change, **c** DAI, and **d** colon length were measured at the indicated time points. **e** Representative H&E, AB-PAS (Scar bars was 100 μm), Arg1, and IL-10 (Scar bars were 50 μm) staining of colon sections. **f** Expression of M2 macrophage markers (Arg1 and IL-10) and M1 macrophage markers (NOS2 and TNF-α) were measured by Western blotting. **g** LPMCs were isolated, stained for CD16/32 (M1 macrophage marker) and CD206 (M2 macrophage marker), and determined by flow cytometry. The ratio of M1 and M2 macrophage was shown at the right panel. **h** Representative immunofluorescence staining of F4/80 (macrophage markers) and CD206 (M2 macrophage markers). Scar bars were 100 μm. Data are presented as means ± SD, n = 6 mice per group. *P < 0.05; **P < 0.01; ***P < 0.001.

The effects of *C. butyricum*-derived EVs on the M2 macrophage polarization were further evaluated by depleting the gut microbiome using an antibiotics blend (Fig. 5a). The DAI score, weight loss, and colon length were not significantly different between the antibiotics+ DSS+ EVs group and the antibiotics+ DSS group (Fig. 5b–d). In addition, H&E and AB-PAS staining demonstrated that *C. butyricum*-derived EVs did not alleviate DSS-induced mucosal necrosis, goblet cell loss, or inflammatory cell infiltration in antibiotic-treated mice. Additionally, in comparison to the antibiotics+ DSS group, the antibiotics+ DSS+ EVs group revealed no significant alterations in histological changes (Fig. 5e).

In the antibiotics+ DSS group, lower expression of Arg1 and IL-10 and elevated expression of NOS2, TNF-α were detected than in the Control group. However, after receiving antibiotic clearance, the expression levels of the above M1 (NOS2 and TNF-α) or M2 (Arg1 and IL-10) markers were not substantially different between the antibiotics+ DSS and antibiotics+ DSS+ EVs groups (Fig. 5e–f). Furthermore, the difference in the proportion of M1 (F4/80+ CD16/32+) and M2 (F4/80+ CD206+) cells in the LP in the antibiotics+ DSS group and the antibiotics+ DSS+ EVs group was insignificant (Fig. 5g), which was further confirmed by immunofluorescence staining for F4/80 and CD206 (Fig. 5h). These results indicated that the altered gut microbiota following *C. butyricum*-derived EVs administration was responsible for the improved M1/M2 equilibrium in the intestinal mucosa. In summary, C*. butyricum*-derived EVs regulated macrophage repolarization in a gut microbiota-dependent manner.

### *C. butyricum*-derived EVs fecal transplantation improved UC by improving M2 macrophage polarization

**Fig.6:**
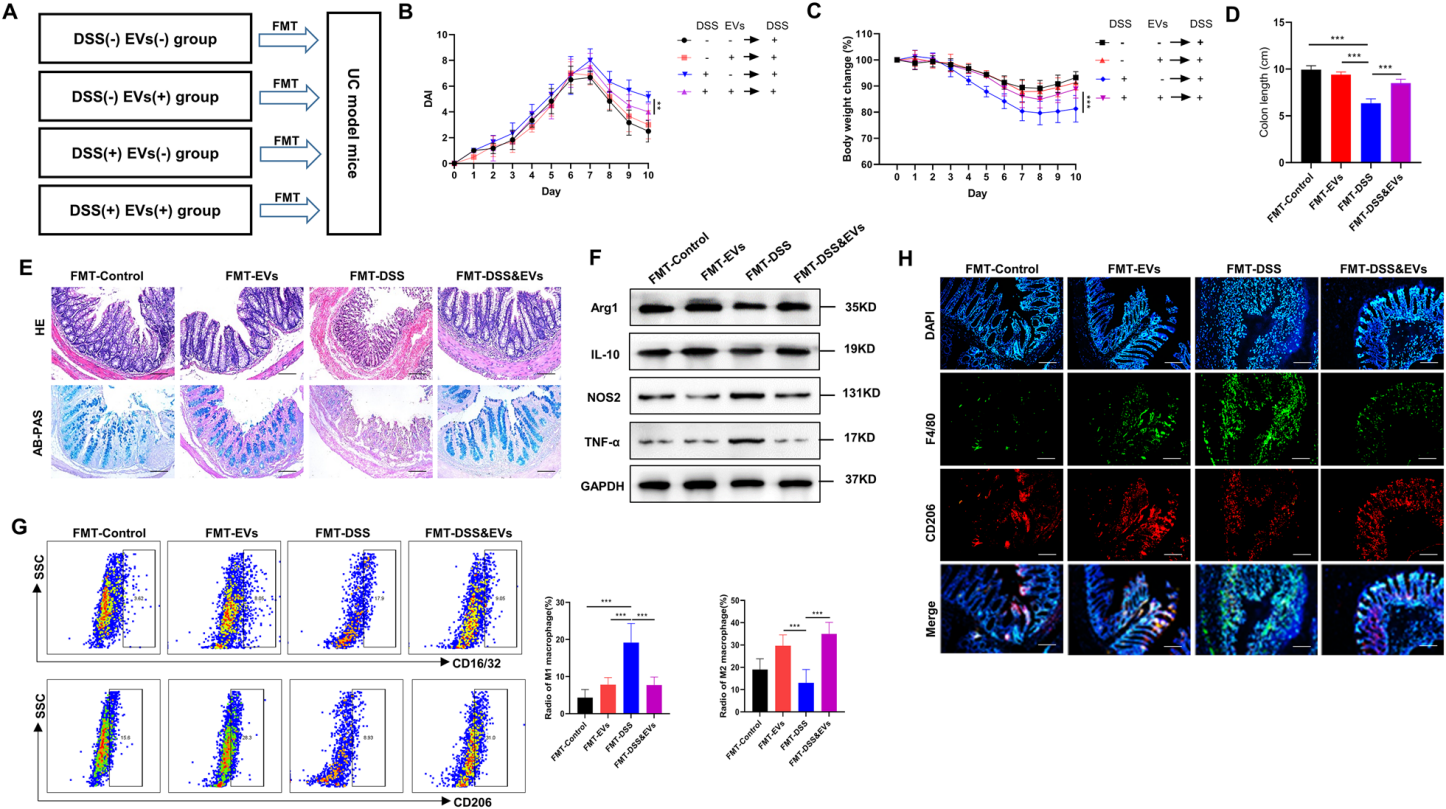
*C. butyricum*-derived EVs faecal transplantation improved UC by improving M1/M2 balance. **a** Donor mice were randomly divided into 4 groups: DSS−EVs−, DSS−EVs+ , DSS+ EVs−, and DSS+ EVs+ . Fecal sample from donor mice was transplanted to DSS-induced colitic mice. **b** DAI, **c** body weight change, and **d** colon length was measured. **e** Representative photomicrographs of H&E and AB-PAS stained of colon tissues. Scar bars were 100 μm. **f** Colonic mucosa was analyzed by Western blotting to detect the expression of M2 macrophage markers (Arg1 and IL-10) and M1 macrophage markers (NOS2 and TNF-α). **g** Flow cytometry staining of LPMCs for CD16/32 (M1 macrophage marker) and CD206 (M2 macrophage marker). The ratio of M1 and M2 macrophage was shown at the right panel. **h** Representative immunofluorescence staining of F4/80 (macrophage markers) and CD206 (M2 macrophage markers). Scar bars were 100 μm. Data are presented as means ± SD, n = 6 mice per group. *P < 0.05; **P < 0.01; ***P < 0.001

Modification of gut microbiota by faecal microbiota transplantation (FMT) has shown as a promising tool in the treatment of UC. We transplanted the gut microbiome of DSS + EVs−, DSS−EVs−, DSS+ EVs+ , and DSS−EVs+ into mice with DSS-induced UC (Fig. 6a). Reduced DAI score, weight loss, mucosal necrosis, goblet cell damage and inflammatory cell infiltration were observed for the UC mice that were transplanted with faecal matter DSS−EVs+ , DSS-EVs−, and DSS+ EVs+ mice than the mice who received a faecal transplant from DSS+ EVs− mice (Fig. 6a–c, e). Also, faecal transplants from DSS−EVs− and DSS−EVs+ mice resulted in a considerably enhanced colon length compared with DSS+ EVs− mice (Fig. 6d). Additionally, a faecal transplant from DSS−EVs− and DSS−EVs+ resulted in elevated protein expression of Arg1 and IL-10 than from DSS+ EVs− mice. However, downregulated expression of NOS2 and TNF-α was observed in UC mice that were given faecal transplants from DSS−EVs−, DSS−EVs+ , and DSS+ EVs+ mice than from DSS+ EVs− mice **(**Fig. 6f). Fecal transplants from DSS−EVs− and DSS−EVs+ group resulted in lower levels of the cells of M1 (F4/80 + CD16/32 +) and higher levels of M2 (F4/80+ CD206+) cells (also from DSS+ EVs+ group) than from DSS+ EVs− group (Fig. 6g). The immunofluorescence results also showed that in the colonic mucosa of UC mice that processed with a faecal transplant from DSS−EVs−, DSS−EVs+ , and DSS+ EVs+ groups, the number of CD206 positive M2 macrophages was upregulated than from DSS+ EVs− group (Fig. 6h), which was in accord with the previous experiments.

## Discussion

Previous researches have illustrated that gut microbiota performs a vital task in host physiology and immunity [29, 30]. The interaction of complex host-microbiota involves the host sensing of the bacterial metabolites, as well as direct communication with the bacteria. However, usually, bacteria are known to be physically divided from the host by the layer of mucus. Also, studies have shown the differential effects elicited by live and heat-inactivated bacteria, indicating that bacterial membrane recognition involves more than a simple passive interaction [31]. Shen et al. first showed that commensal bacteria could produce EVs, which are membrane-enclosed vesicles released from almost all cell types [32]. They possess the capability of transferring data to other cells and affect their function [10, 14, 32]. However, there is a scarcity of studies on Gram-positive bacterial *C. butyricum*-derived EVs, due to the presence of a thick cell wall, which inhibits the release of *C. butyricum*-derived EVs. In 2009, a study demonstrated the generation of EVs by *Staphylococcus aureus* (Gram-positive bacteria) via TEM and proteomic analyses [33]. Since then, EV production has been studied in other Gram-positive strains, including *Streptococcus pneumoniae, Clostridium perfringens,* and *Bacillus subtilis *[34–36]*.* However, the potential mechanism of action of *C. butyricum*-derived EVs has not been studied. Here, we found that the regulation of M2 macrophages and reverse of the gut microbial dysbiosis performed an essential task in the mechanism of *C. butyricum*-derived EVs in protecting against UC.

The plasticity of macrophages allows the flexibility to alter their phenotypes. A study on acute lung injury-related diseases showed that *Lactobacillus reuteri*-derived EVs minimized the lipopolysaccharide (LPS)-induced pro-inflammatory macrophage response [37]. Another study on the DSS-induced murine colitis model revealed that EVs secreted from symbiont commensals inhibited pro-inflammatory and promoted anti-inflammatory macrophage differentiation and accelerated mucosal repair through recruiting myeloid cells to the wound site [38]. Herein, the role of *C. butyricum*-derived EVs on the alteration of anti-inflammatory macrophages (M2) in UC was explored. The process of M1 macrophage differentiation from M0 macrophages occurs in response to stimulation by either LPS alone or in combination with Th1 cytokines, containing interferon-γ (IFN-γ), granulocyte–macrophage colony-stimulating factor (GM-CSF) and, and is mediated by NOS2 [39]. M1 macrophages inhibit cellular proliferation and damage surrounding tissue by releasing pro-inflammatory cytokines (IL-6, IL-12, IL-1β, TNF-α), NO, and reactive oxygen species (ROS) [40]. On the contrary, M2 macrophages promote cellular proliferation and tissue repair through releasing anti-inflammatory cytokines (TGF-β and IL-10); ornithine and polyamines via the Arg1 pathway; and are associated with upregulated levels of scavenger receptors, DC-SIGN, Dectin-1, and mannose receptors. Also, Th2 cytokines (IL-13 and IL-4) polarize macrophages to favor the M2 type [3, 39]. The transition between the M1 and M2 phenotypes varies along a continuous pro-inflammatory spectrum. However, besides the M1 and M2 phenotypes, an M3 switching phenotype was recently reported to be involved in the development of immune phenomena and disease [41]. Previous studies have revealed a tight regulation of the M1–M2 polarization through multiple signalling pathways and transcriptional/post-transcriptional regulatory pathways, and an imbalance can induce the onset of several types of inflammatory disorders [42]. Re-establishing the M1–M2 equilibrium involves repairing the intestinal homeostasis, which would also provide relief from IBD [3, 42] In our results, we found that *C. butyricum*-derived EVs re-established the M1/M2 balance in mice with DSS-induced UC, and there is no doubt that macrophage polarization will be a novel drug target in clinical practice (Fig. 2).

Previous research has demonstrated that *Lactobacillus*, as part of the human microbiota, suppressed the release of TNF-α and IL-6 by reducing the expression of TNF-alpha-converting enzyme on host cells, which promotes the release of IL-10 [43]. Also, *Fusobacterium* and *Helicobacter* colonize the gastrointestinal tract to induce the differentiation of M1 macrophages in the intestinal LP [6, 44] Thus, we used high throughput screening to study the changes in the composition of the gut microbiota. The DSS-induced changes in the beta diversity of species in the gut microbiota were reflected in the significant distance between each group, except for the DSS+ EVs and the Control  groups, based on the system clustering tree and the PCoA analysis (Fig. 3). *Helicobacter, Escherichia–Shigella, Desulfovibrio* are known to be harmful to the intestinal epithelial cells, and thus, higher levels have been observed during UC [44, 45]. *Lactobacillus, Akkermansia,* and *Eubacterium* can reduce colonic inflammation by improving the integrity of the mucosal barrier [46–49]. This study showed a decrease in the relative abundance of *Helicobacter,* and *Escherichia–Shigella,* respectively, post-treatment with *C. butyricum*-derived EVs in mice with DSS-induced UC. Also, *C. butyricum*-derived EVs treatment induced a considerable increase in *Lactobacillus, Akkermansia *and* Bacteroides* (Fig. 4)*,* which have been associated with SCFAs metabolism [50]. Recent studies have shown that intestinal microbial community composition and bacterial metabolites can significantly improve the outcome of IBD through regulating inflammatory responses [30]. In particular, microbiota-derived SCFAs play an essential role in stabilizing intestinal immune homeostasis through their anti-inflammatory and immune-suppressive functions [30, 51].

Next, we explored the role of gut microbiome in *C. butyricum*-derived EVs induced M2 macrophage polarization in UC by depleting the gut microbiome using an antibiotics blend. Fecal DNA analysis confirmed the depletion of gut microbiota. Reduced impact of *C. butyricum*-derived EVs on UC under these conditions showed that *C. butyricum*-derived EVs hardly induce the polarization of M2 macrophages post-depletion of gut microbiota (Fig. 5). Additionally, faecal matter transplant offers an innovative method to analyze the effects of products of bacteria on gut microbiota. A study showed that reduced high fat diet-induced obesity and insulin resistance in mice who had received faecal transplants from donor mice taking blueberry proanthocyanidins and anthocyanins, which implied the role of gut microbiota in blueberry polyphenols-induced reduction in obesity and insulin resistance [52]. Recent evidence has also led to bacterial extracellular RNAs being proposed as potential candidates for microbe-microbe and microbe-host inter-communication tools as they can be delivered to recipient cells and potentially regulate the expression of genes involved in controlling bacterial growth, biofilm formation and metabolic function [10, 14]. However, research findings on the relevance of interactions between EVs and microbiota in the context of human diseases are still in their infancy. Here, we performed faecal transplantation and found reduced UC and modified M1/M2 equilibrium in mice which received a faecal transplant from *C. butyricum*-derived EVs-treated mice, and the positive effect seemed to be related to the M2 macrophages repolarization (Fig. 6). Our findings indicated that the altered faecal microbiota following *C. butyricum*-derived EVs administration resulted in alleviated colon inflammation, and improved the M1/M2 macrophage balance during IBD progression.

In this study, we first found that *C. butyricum*-derived EVs promoted M2 macrophage polarization and regulated the homeostasis of gut microbiota in mice with DSS-induced UC. Next, the depletion of gut microbiota caused *C. butyricum*-derived EVs to fail to induce the polarization of M2 macrophages, while faecal transplant from donor mice who received *C. butyricum*-derived EVs restored the disturbed M1/M2 balance in recipient mice. Thus, this study highlighted the interaction between *C. butyricum*-derived EVs and gut microbiota in improving UC by polarizing M2 macrophages, and the use of bacteria-derived EVs with probiotic properties may provide a novel approach for the treatment of UC.

## Supplementary Information

**Additional file 1: Figure S1.** Effects of *C. butyricum*-derived EVs on overall structural modulation of gut microbiota in DSS-induced colitis mice. **A** Shannon index calculated for all samples, the differences in the Shannon indexes between the groups were not statistically significant.

## Data Availability

The authors will make available the raw data supporting the conclusions of this article upon reasonable request.

## Abbreviations

EVs: Extracellular vesicles
*C. butyricum*: *Clostridium butyricum*
DSS: Dextran sulfate sodium
IBD: Inflammatory bowel disease
UC: Ulcerative colitis
CD: Crohn’s disease
TNF-α: Tumor necrosis factor-α
EN: Enteral nutrition
AIEC: Adherent-invasive *Escherichia-Shigella*
NTA: Nanoparticle tracking analysis
CBM588: *Clostridium butyricum* Strain MIYAIRI 588
DAI: Disease activity index
NOS2: Inducible nitric oxide synthase
Arg1: Arginase 1
SCFAs: Short-chain fatty acids
ANOVA: A one-way analysis of variance
IFN-γ: Interferon-γ
GM-CSF: Granulocyte–macrophage colony-stimulating factor
ROS: Reactive oxygen species
LP: Lamina propria
