# Supplementary figures and images for: Commensal bacteria-derived extracellular vesicles suppress ulcerative colitis through regulating the macrophages polarization and remodeling the gut microbiota
Source: Microb Cell Fact. 2022 May 16;21:88. doi: 10.1186/s12934-022-01812-6 (PMC9109417; doi:10.1186/s12934-022-01812-6)

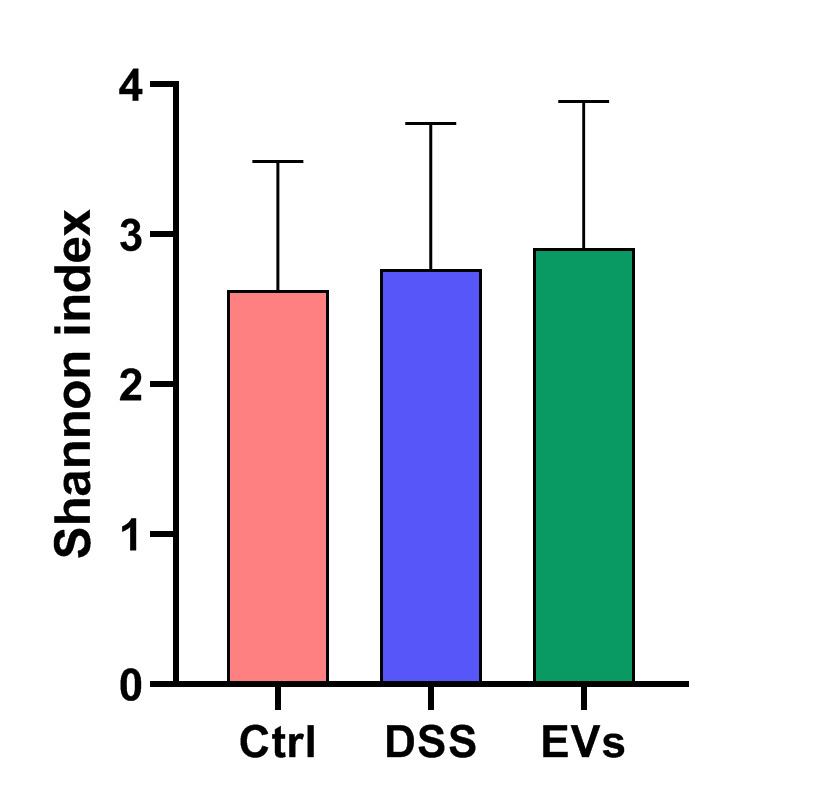

Supplement: Supplementary file 1 — Additional file 1: Figure S1. Effects of C. butyricum-derived EVs on overall structural modulation of gut microbiota in DSS-induced colitis mice. A Shannon index calculated for all samples, the differences in the Shannon indexes between the groups were not statistically significant. [file 12934_2022_1812_MOESM1_ESM.tif]
